# Transplant Trial Watch

**DOI:** 10.3389/ti.2022.10307

**Published:** 2022-03-15

**Authors:** Simon R Knight

**Affiliations:** ^1^ Oxford Transplant Centre, Churchill Hospital, Oxford, United Kingdom; ^2^ Centre for Evidence in Transplantation, Nuffield Department of Surgical Sciences, University of Oxford, Oxford, United Kingdom

**Keywords:** liver transplantation, immunosuppression, lung transplantation, induction, pediatric patients

**Randomised Controlled Trial 1**

**Early Everolimus-Facilitated Reduced Tacrolimus in Liver Transplantation: Results from the Randomized HEPHAISTOS Trial**

*by Nashan, B., et al. Liver Transplantation [record in progress].*

**Randomised Controlled Trial 2**

**CTOTC-08: A Multicenter Randomized Controlled Trial of Rituximab Induction to Reduce Antibody Development and Improve Outcomes in Pediatric Lung Transplant Recipients**

*by Sweet, S. C., et al. American Journal of Transplantation [record in progress].*

To keep the transplantation community informed about recently published level 1 evidence in organ transplantation ESOT and the Centre for Evidence in Transplantation have developed the Transplant Trial Watch. The Transplant Trial Watch is a monthly overview of 10 new randomised controlled trials (RCTs) and systematic reviews. This page of Transplant International offers commentaries on methodological issues and clinical implications on two articles of particular interest from the CET Transplant Trial Watch monthly selection. For all high quality evidence in solid organ transplantation, visit the Transplant Library: www.transplantlibrary.com.

RANDOMISED CONTROLLED TRIAL 1 Early everolimus-facilitated reduced tacrolimus in liver transplantation: Results from the randomized HEPHAISTOS trial

*by Nashan, B., et al. Liver Transplantation [record in progress].*

## Aims

This study aimed to investigate the outcomes related to early initiation of everolimus-facilitated reduced-exposure tacrolimus (EVR + rTAC) in *de novo* liver transplant patients.

## Interventions

Participants were randomised to either the group that received EVR + rTAC or the group receiving standard-exposure tacrolimus (sTAC) with steroids.

## Participants

333 *de novo* liver transplant recipients.

## Outcomes

The primary outcome was renal function. The secondary outcomes included death, graft loss, acute rejection (AR), treated AR or treated biopsy-proven acute rejection (tBPAR), assessed as composite or individual components at 12 months posttransplant.

## Follow-up

12 months.

## CET Conclusion

The HEPHAISTOS superiority trial compared everolimus plus reduced exposure tacrolimus versus everolimus with standard exposure tacrolimus in *de novo* liver transplant recipients. The multicentre, German study randomised recipients 7–21 days posttransplant using a validated system that automates random assignment. The power analysis indicated that 105 patients in each group were needed, which was adjusted to 165 patients per group to allow for dropouts. The study randomised 333 patients and the primary full-analysis set, which included all randomised patients who received at least one dose of the study drug, found no statistically significant difference in eGFR at 12 months between groups. A statistically significant difference between groups in eGFR was found for the per-protocol and on-treatment analyses. The composite efficacy-endpoint of graft loss, death or treated BPAR was similar between groups. Treatment-emergent (serious) adverse events were similar between groups but there were more adverse events leading to study drug interruption or adjustment in the reduced exposure tacrolimus group.

## Jadad Score

3.

## Data Analysis

Intention-to-treat analysis.

## Allocation Concealment

Yes.

## Trial Registration

ClinicalTrials.gov, NCT01551212; EudraCT, 2011-003118-17.

## Funding Source

Industry funded.

RANDOMISED CONTROLLED TRIAL 2 CTOTC-08: A Multicenter Randomized Controlled Trial of Rituximab Induction to Reduce Antibody Development and Improve Outcomes in Pediatric Lung Transplant Recipients

*by Sweet, S. C., et al. American Journal of Transplantation [record in progress].*

## Aims

The aim of this study was to investigate whether rituximab in addition to rabbit anti-thymocyte globulin induction was effective in reducing the development of *de novo* donor-specific human leukocyte antigen antibodies (DSA) and improve outcomes, in paediatric lung transplant recipients.

## Interventions

Participants were randomly assigned to either the rituximab group or the placebo group.

## Participants

27 paediatric lung transplant patients.

## Outcomes

The primary outcome was a composite of chronic allograft dysfunction, listing for re-transplant or death. The secondary outcomes were the incidence of primary graft dysfunction, antibody-mediated rejection and acute cellular rejection.

## Follow-up

24 months.

## CET Conclusions

This is a good quality randomised controlled trial in paediatric lung transplantation. The study was double-blinded and conducted in multiple centres. Patients were randomised to either standard immune induction with ATG (plus placebo) or to ATG and Rituximab. The primary outcome was composite graft dysfunction, death or re-listing. Unfortunately, only 11 subjects met criteria for the composite primary outcome, so the study was underpowered to demonstrate all but the most drastic of differences between the study arms. Whilst there was no significant difference in the primary outcome, there was a significantly lower generation of *de novo* DSA in the Rituximab arm (21% vs. 73%). There was no significant difference in adverse event rates. A much larger study, and with longer follow up, is required.

## Jadad Score

5.

## Data Analysis

Intention-to-treat analysis.

## Allocation Concealment

Yes

## Trial Registration

ClinicalTrials.gov, NCT02266888.

## Funding Source

Non-Industry funded.

## Clinical Impact Summary

Most current induction immunosuppression strategies focus on T-cell inactivation or depletion. B-cell activation and donor-specific antibody production also play an important role in allograft damage, which has led to interest in the use of B-cell depleting therapies such as rituximab as induction agents following solid organ transplantation.

In a recent publication in the American Journal of Transplantation, Sweet et al. report a multicentre randomised-controlled trial using rituximab as induction therapy in paediatric lung transplant recipients (1). The study is well designed, with double blinding and allocation concealment ensured by use of placebo and centralised web-based randomisation. Unfortunately, the study failed to recruit the required target sample within the funding time-frame, resulting in a loss of power and shorter follow-up than initially planned. Perhaps as a result, no difference in the primary clinical endpoint [a composite of death, bronchiolitis obliterans syndrome (BOS) and relisting] was seen. However, there was a significantly lower incidence of *de novo* donor specific antibodies (DSA) in the rituximab-treated group, leading the authors to cautiously claim some evidence of benefit.

Whilst it is difficult to draw firm conclusions from an underpowered study, the suggestion of benefit seen in this study is at odds with previous studies in renal and cardiac transplantation. A systematic review of studies in renal transplantation from our own group in 2014 found no clear evidence of benefit to rituximab induction across a small number of studies (2). The authors of the current study postulate that this may be due to a lack of T-cell depleting induction in these earlier studies. Rituximab also depletes regulatory B-cells, and this loss of regulation in the presence of donor-reactive T-cells may increase the risk of T-cell mediated rejection. Combination of B- and T-cell depletion is proposed to overcome this.

One specific area of concern, perhaps not apparent in the current paediatric study, is the impact of rituximab therapy on the risk of cardiovascular disease. Previous studies in both renal transplantation and cardiac transplantation have suggested increased risk of cardiovascular mortality and graft vessel disease, possibly related to the role of B-regulatory cells in atheroprotection (3, 4). Any future studies, especially in adult populations, would need to collect these outcomes and ensure long-enough follow-up to adequately assess the impact on cardiac disease.

Overall, the study does provide some interesting data suggestive of a potential role of B-cell depletion in conjunction with T-cell depleting induction in the reduction of DSA formation and subsequent chronic allograft damage. Further, well-powered studies in adult populations will need to focus on the long-term safety of such a strategy.
